# Comparisons of GnRH antagonist protocol versus GnRH agonist long protocol in patients with normal ovarian reserve: A systematic review and meta-analysis

**DOI:** 10.1371/journal.pone.0175985

**Published:** 2017-04-24

**Authors:** Ruolin Wang, Shouren Lin, Yong Wang, Weiping Qian, Liang Zhou

**Affiliations:** 1 Reproductive Medical Center, Peking University Shenzhen Hospital, Shenzhen, China; 2 Medical College of Shantou University, Shantou, China; Institute of Zoology Chinese Academy of Sciences, CHINA

## Abstract

**Objective:**

To evaluate the effectiveness and safety of gonadotropin-releasing hormone antagonist (GnRH-ant) protocol and gonadotropin-releasing hormone agonist (GnRH-a) long protocol in patients with normal ovarian reserve.

**Methods:**

We searched the PubMed (1992–2016), Cochrane Library (1999–2016), Web of Science (1950–2016), Chinese Biomedical Database (CBM, 1979–2016), and China National Knowledge Infrastructure (CNKI, 1994–2016). Any randomized controlled trials (RCTs) that compared GnRH-ant protocol and GnRH-a long protocol in patients with normal ovarian reserve were included, and data were extracted independently by two reviewers. The meta-analysis was performed by Revman 5.3 software.

**Results:**

Twenty-nine RCTs (6399 patients) were included in this meta-analysis. Stimulation days (mean difference (MD) [95% confidence interval (CI)] = -0.8 [-1.36, -0.23], P = 0.006), gonadotrophin (Gn) dosage (MD [95% CI] = -3.52 [-5.56, -1.48], P = 0.0007), estradiol (E2) level on the day of human chorionic gonadotrophin (HCG) administration (MD [95% CI] = -365.49 [-532.93, -198.05], P<0.0001), the number of oocytes retrieved (MD [95% CI] = -1.41 [-1.84, -0.99], P<0.00001), the embryos obtained (MD [95% CI] = -0.99 [-1.38, -0.59], P<0.00001), incidence of ovarian hyperstimulation syndrome (OHSS) (OR [95% CI] = 0.69 [0.57, 0.83], P<0.0001) were statistically significantly lower in GnRH-ant protocol than GnRH-a long protocol. However, the clinical pregnancy rate (OR [95% CI] = 0.90 [0.80, 1.01], P = 0.08), ongoing pregnancy rate (OR [95% CI] = 0.88 [0.77, 1.00], P = 0.05), live birth rate (OR [95% CI] = 0.95 [0.74, 1.09], P = 0.27), miscarriage rate (OR [95% CI] = 0.98 [0.69, 1.40], P = 0.93), and cycle cancellation rate (OR [95% CI] = 0.86 [0.52, 1.44], P = 0.57) showed no significant differences between the two groups.

**Conclusion:**

GnRH-ant protocol substantially decreased the incidence of OHSS without influencing the pregnancy rate and live birth rate compared to GnRH-a long protocol among patients with normal ovarian reserve.

## Introduction

Since gonadotropin-releasing hormone agonist (GnRH-a) was developed in the 1980s [1], it has played an important role in controlled ovarian hyperstimulation (COH) among patients who are undergoing assisted reproductive technology (ART). The advantage of GnRH agonist is to prevent premature luteinizing hormone (LH) surge, thereby increasing the number of retrieved oocytes and pregnancy rates and decreasing the number of cycle cancellations [2, 3]. These advantages, however, may lead to ovarian hyperstimulation syndrome (OHSS) or other side effects [4].

GnRH antagonist (GnRH-ant), which was discovered in the 1990s, can competitively block GnRH receptors and cause rapid suppression of Gn release [5]. This protocol has fewer complications and is more convenient for patients because of the shorter treatment time and fewer injections [6]. However, its effectiveness is still debated.

Multiple studies, including meta-analyses and randomized controlled trials (RCTs), of the GnRH-a protocol and GnRH-ant protocol on pregnancy rate and live birth rate have yielded controversial findings [6–8]. A 2006 Cochrane systematic review of 27 RCTs showed that GnRH-ant protocol has a significantly lower clinical pregnancy rate and live birth rates than those in GnRH-a long protocol, while the incidence of OHSS is significantly lower in GnRH-ant protocol [9]. However, a 2011 Cochrane systematic review of 45 RCTs found that there was no significant difference in the live birth rates between the GnRH-a and GnRH-ant groups [10]. A recent Cochrane systematic review of 73 RCTs in 2016 also concluded that these two protocols have equivalent live birth rates, and GnRH-ant protocol has a lower incidence of OHSS [11].

The finding that GnRH-ant protocol reduces the pregnancy rate may result from the fact that some centers only choose GnRH-ant protocol as their second treatment option in COH, or use it to treat the patients with an unfavorable prognosis, such as repeated implantation failures, older patients, and low responders [12]. This study’s purpose is to determine the effectiveness and safety of GnRH-a long protocol and GnRH-ant protocol among patients with normal ovarian reserve to unify the influencing factors.

## Materials and methods

### Search strategy

“GnRH agonist”, “GnRHa”, “GnRH antagonist”, “GnRH-ant”, “GnRHA”, “randomized controlled trial”, “RCT”, and “Normal ovarian reserve” were used as the keywords for the literature searches in the PubMed (1992–2016), Cochrane Library (1999–2016), Web of Science (1950–2016), Chinese Biomedical Database (CBM,1979–2016), and China National Knowledge Infrastructure (CNKI,1994–2016) databases. The retrieval time was from the first publication of the journal to the end of December 2016. References included in the studies were also searched.

### Inclusion and exclusion criteria

Inclusion criteria were RCTs that compared the effectiveness and safety of GnRH-a long protocol and GnRH-ant protocol in patients with normal ovarian reserve. Exclusion criteria included failure to report appropriate randomized procedures, classification of participants as low or high ovarian response or endometriosis, and unclear or inappropriate outcomes. Editorials, letters to the editor, review articles, case reports and animal experimental studies were also excluded.

### Data extraction and quality assessment

Studies were screened by two reviewers (R.W. and Y.W.) independently, and any disagreement was settled by consensus. First, the title and abstract of each study was read carefully to exclude the studies that clearly did not meet the inclusion criteria. Then, the full text of the remaining studies was read to determine which studies would be included in this study.

The quality assessment of RCTs was compiled using Cochrane’s risk of bias tool [13], which included sequence generation, allocation concealment blinding of participants, personnel and outcome assessors, incomplete outcome data, selective outcome reporting, and other sources of bias.

### Outcome measures

The main efficacy outcome measures included the clinical pregnancy rate (defined as the presence of a gestational sac on ultrasound or gestational sac with fetal heart tones), the ongoing pregnancy rate (determined as pregnancies with over 12 weeks of gestation), and the live birth rate. The secondary efficacy outcome measures were ovarian stimulation outcomes, which included stimulation days, gonadotropin dosage, endometrial thickness and estradiol (E2) level on the day of human chorionic gonadotrophin (HCG) administration, the number of oocytes retrieved, and the embryos obtained. The safety outcome measures included the incidence of OHSS, the miscarriage rate, and the cycle cancellation rate.

### Statistical analysis

All statistical analyses were performed using Revman 5.3 software. Dichotomous outcomes were expressed as odds ratios (ORs) with 95% confidence intervals (CIs). Continuous variables were expressed as weighted mean differences (MDs) with 95% CIs. Heterogeneity was evaluated using the Q-test and I^2^-index values, and reported for each outcome as a P-value and percentage, respectively. If heterogeneity was adopted (I^2^<25% or >50% with P>0.1), meta-analysis used a fixed-effects model. Otherwise (I^2^≥50% or I^2^>25% with P≤0.1), meta-analysis used a random-effects model. Sensitivity analysis was used to determine the stability of the results. Begg’s funnel plot and Begg’s test were used to assess publication bias by STATA software (version 12.0, Stata Corp).

## Results

### Eligible studies

**Fig 1:**
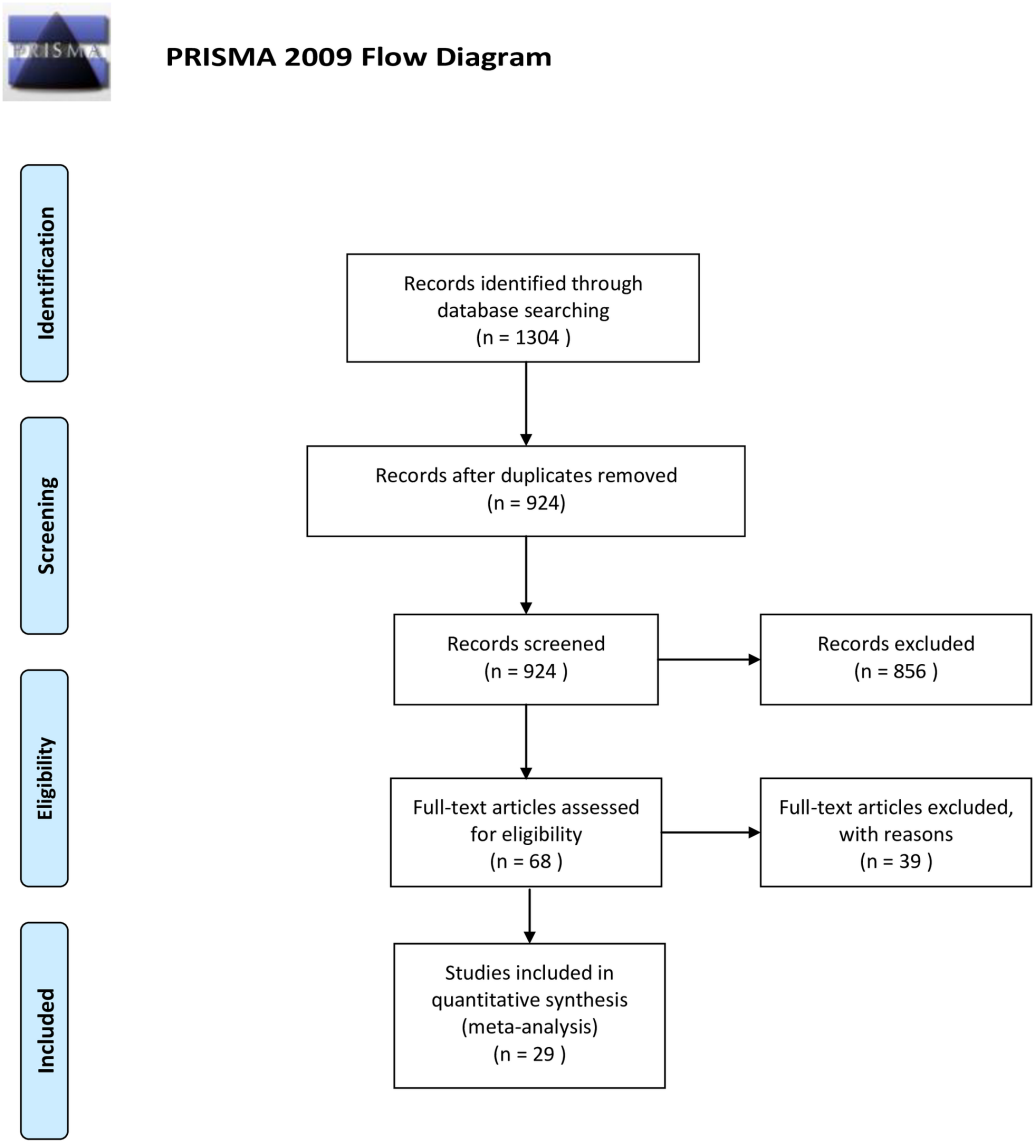
Literature screening flow diagram. *From*: Moher D, Liberati A, Tetzlaff J, Altman DG, The PRISMA Group (2009). *P*referred *R*eporting *I*tems for *S*ystemetic Reviews and *M*eta-*A*nalyses: The PRISMA Statement. PLoS Med 6(7): e1000097. doi:10.1371/journal.pmed1000097.** For more information, visit www.prisma-statement.org**.

The initial literature search yielded 1,304 studies. Screening of the titles and abstracts resulted in 68 published articles that could possibly compare the GnRH-a long protocol and GnRH-ant protocol in patients with normal ovarian reserve. There is no official definition of normal ovarian reserve. Therefore, the studies included in our article mainly depended on a consensus, which met any two of the following criteria: Age < 40 years, normal menstrual cycle, basal FSH ≤ 10 IU/L, basal E2 < 60 pg/ml, AFC > 5, previous IVF attempts < 3, no previous poor response/OHSS history, no polycystic ovary syndrome (PCOS) / severe endometriosis. After reading the full papers, only 29 studies (6399 patients) finally met our inclusion criteria [8, 14–41]. Although the cited studies used different drugs and doses for COH, they all belong to either GnRH-ant protocol or GnRH-a protocol, so we included these studies in a general way. The literature screening process and the results are shown in Fig 1, and the basic characteristics of the included papers are shown in Table 1. The quality assessment results are shown in S1 Table.

**Table 1:** Characteristics of included studies.

Study	Inclusion Criteria	No. of patients		Protocol		Gn type & initial dosage(IU/d)
		GnRH-ant	GnRH-a	GnRH-ant	GnRH-a	
Albano et al. 2000	Age ≤39 y, basal FSH ≤10IU/L, regular menstrual cycle, previous IVF cycle ≤3	198	95	Multiple dose (cetrorelix)	Long, multiple dose (buserelin)	HMG 150
European Orgalutran. 2000	Age ≤39 y, BMI 18–29 kg/m^2^, regular menstrual cycle	486	244	Multiple dose (ganirelix)	Long, multiple dose (buserelin)	rFSH 150
Olivennes et al. 2000	Age ≤39 y, basal FSH≤10IU/L, normal menstrual cycle, previous IVF attempts≤3	126	43	Single dose (cetrorelix)	Long, single dose (triptorelin)	HMG unclear
European and Middle East Orgalutration. 2001	Age <39 y, BMI 18–29 kg/m^2^, regular menstrual cycle	236	119	Multiple dose (ganirelix)	Long, single dose (triptorelin)	rFSH 150
Fluker et al. 2001	Age <39 y, basal FSH <10 IU/L, basal LH <10 IU/L, BMI 18–29 kg/m^2^, regular menstrual cycle	205	108	Multiple dose (ganirelix)	Long, multiple dose (leuprolide)	rFSH 225
Hohmann et al.2003	Age <39 y, BMI 19–29 kg/m^2^, regular menstrual cycle, previous IVF attempts ≤3, no previous poor response/OHSS history	97	45	Multiple dose (cetrorelix)	Long, single dose (triptorelin)	rFSH 150
Check et al. 2004	Unclear	30	30	Multiple dose (ganirelix)	Long, multiple dose (leuprolide)	rFSH300 or rFSH150+ HMG150
Loutradis et al. 2004	Age <39 y, regular menstrual cycle, no previous poor response history	58	58	Multiple dose (cetrorelix)	Long, multiple dose (triptorelin)	rFSH 225
Sauer et al. 2004	Age ≤39 y, BMI <35 kg/m^2^, regular menstrual cycle, FSH level in normal range	25	25	Single dose (cetrorelix)	Long, multiple dose (leuprolide)	rFSH 225
Badrawi et al. 2005	Age ≤39 y, basal FSH <10 IU/L, regular menstrual cycle	50	50	Multiple dose (ganirelix)	Long, multiple dose (buserelin)	HMG 225
Barmat et al. 2005	Age <39 y,BMI 19–32 kg/m^2^, basal FSH≤10 IU/L, E2<60 pg/ml, AFC >5, regular menstrual cycle, previous failed IVF cycle≤1, no previous poor response history	40	40	Multiple dose (ganirelix)	Long, multiple dose (leuprolide)	rFSH300
Lee et al. 2005	Age ≤39 y, BMI 18–29 kg/m^2^, basal FSH ≤10 IU/L, regular menstrual cycle, no previous poor response history	20	20	Multiple dose (cetrorelix)	Long, multiple dose (buserelin)	HMG 225
Xavier et al. 2005	Age ≤39 y, basal FSH ≤10 IU/L, previous IVF attempts ≤3	66	65	Multiple dose (cetrorelix)	Long, multiple dose (buserelin)	rFSH 150–450
Ferrari et al. 2006	Age ≤39 y, BMI 20–25 kg/m^2^, basal FSH< 10 IU/L, basal E2≤45 IU/L, regular menstrual cycle	30	30	Multiple dose (cetrorelix)	Long, multiple dose (leuprolide)	rFSH 225
Friedler et al. 2006	Age <35 y, basal FSH≤10 IU/L	37	36	Multiple dose (ganirelix)	Long, multiple dose (buserelin)	rFSH 225
Rombauts et al. 2006	Age ≤39 y, BMI 18–29 kg/m^2^, normal menstrual cycle, previous failed IVF cycle ≤3, no previous poor response history	234	117	Multiple dose (ganirelix)	Long, multiple dose (nafarelin)	rFSH 200
Serafini et al. 2006	Age ≤39 y, basal FSH≤15 IU/L, basal E2≤60 IU/L, previous IVF attempts <3, no previous poor response history	217	106	Multiple dose (cetrorelix)	Long, multiple dose (leuprolide)	rFSH 150–300
Baart et al. 2007	Age <38 y, BMI 19–29 kg/m^2^, regular menstrual cycle, no previous IVF attempt	67	44	Multiple dose (orgalutran)	Long, multiple dose (triptorelin)	rFSH 150/225
Heijnen et al. 2007	Age <38 y, BMI 18–28 kg/m^2^, regular menstrual cycle, no previous IVF attempt	205	199	Unclear	Long, unclear	Unclear
Hsieh et al. 2008	Age ≤39 y, body weight of 40–70 kg	86	58	Multiple dose (cetrorelix)	Long, multiple dose (leuprolide)	rFSH 150–225
Moraloglu et al. 2008	Age ≤38 y, BMI <30 kg/m^2^, basal FSH <10 IU/L, previous IVF attempts ≤3, no previous poor response history, no PCOS	45	48	Multiple dose (cetrorelix)	Long, multiple dose (leuprolide)	rFSH 225
Depalo et al. 2009	Age≤42 y, basal FSH <10 IU/L, previous IVF attempts≤3, no PCOS/ severe endometriosis	67	69	Multiple dose (cetrorelix)	Long, multiple dose (leuprolide)	rFSH 225
Ye et al. 2009	Age ≤35 y, BMI 18–25 kg/m^2^, previous IVF attempts <3, no previous poor response history, normal-ovulatory cycles	109	111	Multiple dose (cetrorelix)	Long, multiple dose (leuprolide)	rFSH 225
Firouzabadi et al. 2010	Age <35 y, basal FSH <10 IU/L, no previous IVF attempt	118	107	Multiple dose (ganirelix)	Long, multiple dose (buserelin)	rFSH 150–225
Papanikolaou et al. 2012	Age <39 y, basal FSH <12 IU/L, previous IVF attempts <3,	96	94	Multiple dose (ganirelix/ cetrorelix)	Long, multiple dose (buserelin)	rFSH 150–300
Qiao et al. 2012	Age ≤35 y, BMI 18–29 kg/m^2^, normal menstrual cycle	113	120	Multiple dose (ganirelix)	Long, multiple dose (triptorelin)	rFSH unclear
Rabati et al. 2012	basal FSH ≤10 IU/L, first time of ART	69	67	Multiple dose (cetrorelix)	Long, multiple dose (buserelin)	rFSH 75
Hershko et al. 2015	Age <37 y, previous failed IVF cycles≤3, without ovulatory factor(WHO I-III)	31	29	Multiple dose (cetrorelix)	Long, multiple dose (triptorelin)	rFSH /HMG
Toftager et al. 2016	Age <40 y, previous IVF attempts <3	534	516	Multiple dose (ganirelix)	Long, multiple dose (nafarelin)	rFSH 150/225

### Main outcome measures of effectiveness

#### The clinical pregnancy rate

**Fig 2:**
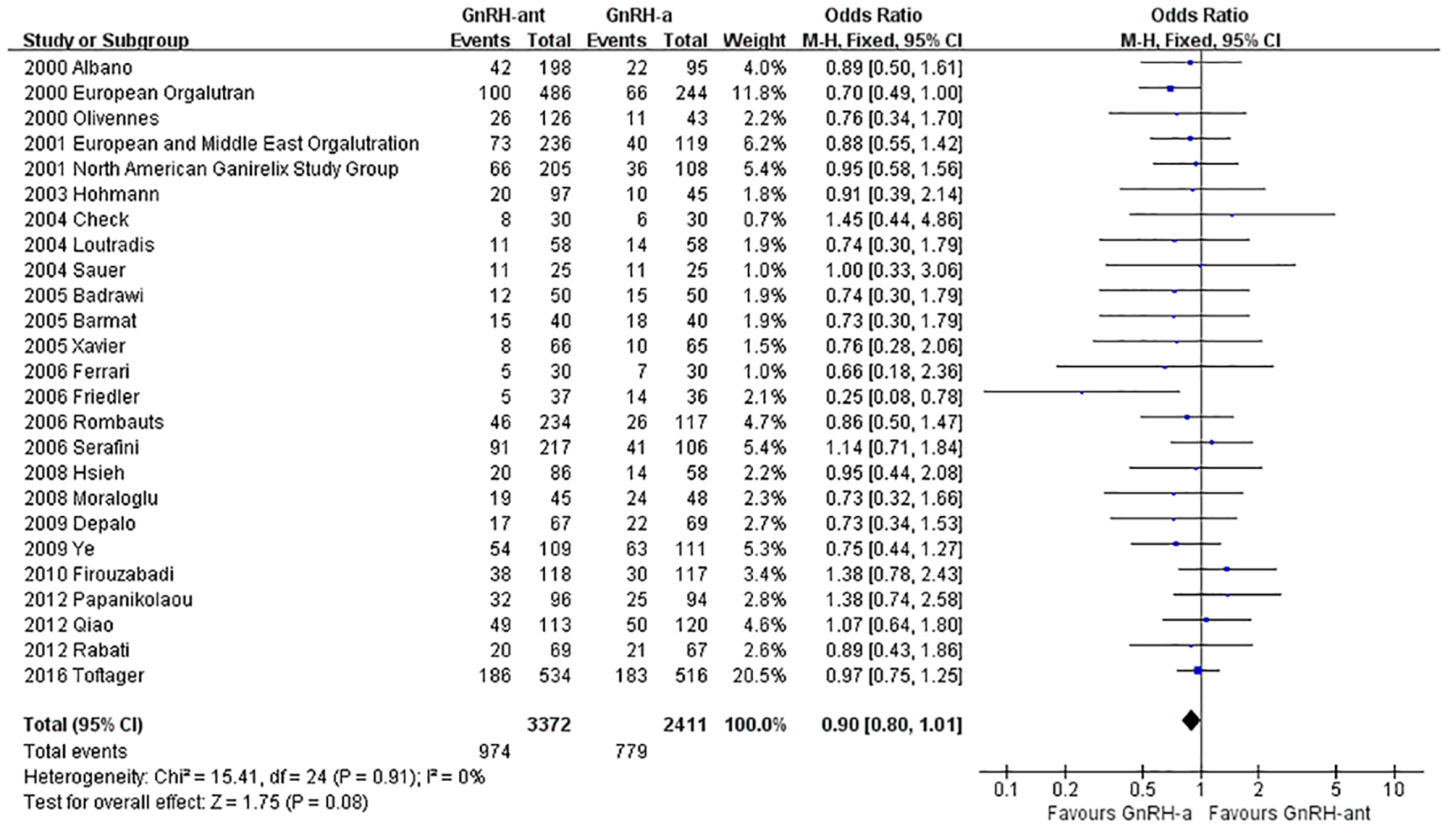
Forest plot comparing the clinical pregnancy rate per woman randomized between the GnRH-ant group and the GnRH-a long-protocol group.

A total of 25 studies [8, 14–23, 25–29, 32–39, 41](5,814 cases) were included in the clinical pregnancy rate meta-analysis. There was no heterogeneity (P = 0.91, I^2^ = 0%) among the trials; therefore, the fixed-effects model was used for the meta-analysis. The results indicated that there was no statistically significant difference in the clinical pregnancy rate between the GnRH-ant group and the GnRH-a long-protocol group (OR [95% CI] = 0.90 [0.80, 1.01], P = 0.08, Fig 2).

#### The ongoing pregnancy rate

**Fig 3:**
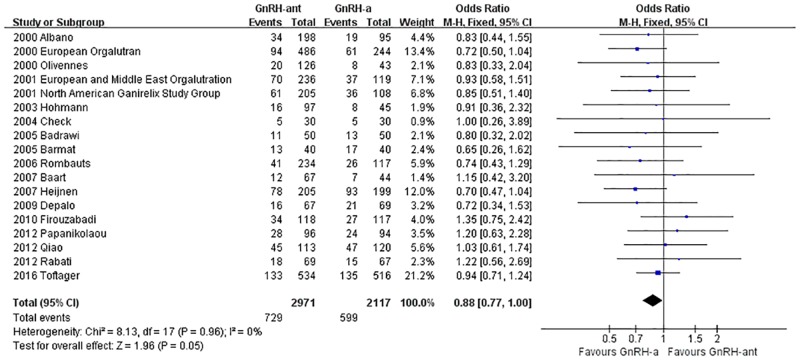
Forest plot comparing the ongoing pregnancy rate per woman randomized between the GnRH-ant group and the GnRH-a long-protocol group.

A total of 18 studies [8, 14–20, 23, 28, 30, 31, 34, 36–39, 41](5,119 cases) were included in the ongoing pregnancy rate meta-analysis. There was no heterogeneity (P = 0.96, I^2^ = 0%) among the trials; therefore, the fixed-effects model was used for the meta-analysis. The results indicated that there was no statistically significant difference in the ongoing pregnancy rate between the GnRH-ant group and the GnRH-a long-protocol group (OR [95% CI] = 0.88 [0.77, 1.00], P = 0.05, Fig 3).

#### The live birth rate

**Fig 4:**
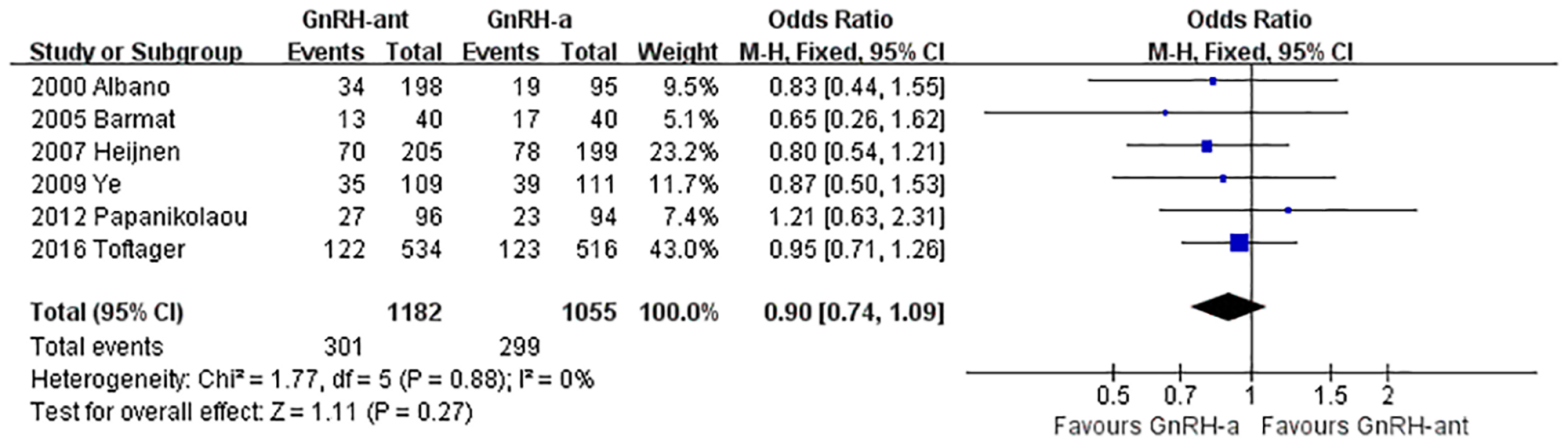
Forest plot comparing the live birth rate per woman randomized between the GnRH-ant group and the GnRH-a long-protocol group.

A total of 6 studies [8, 14, 31, 35, 37, 41](2,237 cases) were included in the live birth rate meta-analysis. There was no heterogeneity (P = 0.88, I^2^ = 0%) among the trials; therefore, the fixed-effects model was used for the meta-analysis. The results indicated that there was no statistically significant difference in the live birth rate between the GnRH-ant group and the GnRH-a long-protocol group (OR [95% CI] = 0.95 [0.74, 1.09], P = 0.27, Fig 4).

### Secondary outcome measures of effectiveness

#### The number of stimulation days

**Fig 5:**
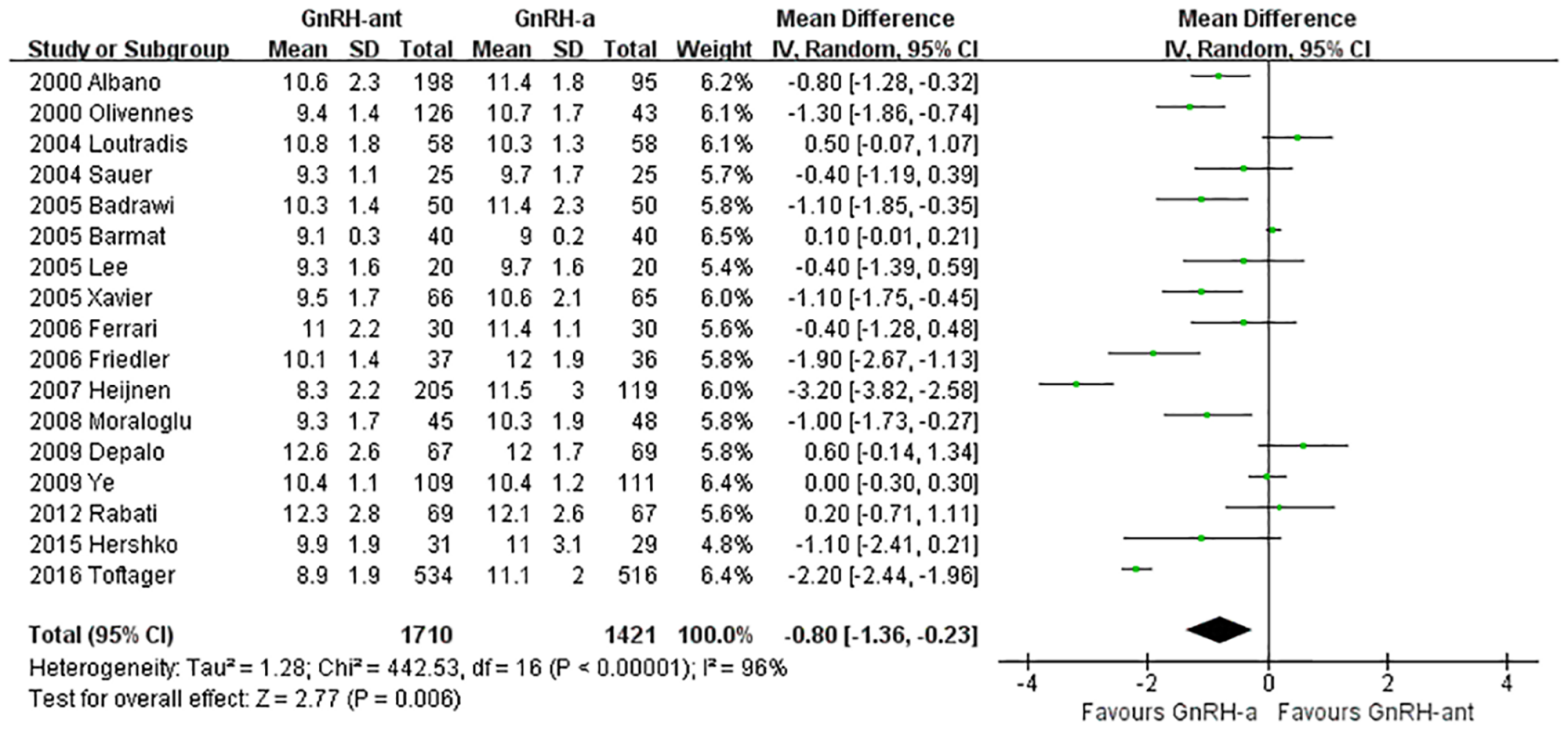
Forest plot comparing the number of stimulation days per woman randomized between the GnRH-ant group and the GnRH-a long-protocol group.

A total of 17 studies [8, 14, 16, 21–27, 31, 33–35, 39–41](3,171 cases) were included in the number of stimulation days meta-analysis. There was heterogeneity (P<0.00001, I^2^ = 96%) among the trials; therefore, the random-effects model was used for the meta-analysis. The results indicated that the stimulation days were statistically significantly fewer in the GnRH-ant group than in the GnRH-a long-protocol group (MD [95% CI] = -0.8 [-1.36, -0.23], P = 0.006, Fig 5).

#### Gn dosage

**Fig 6:**
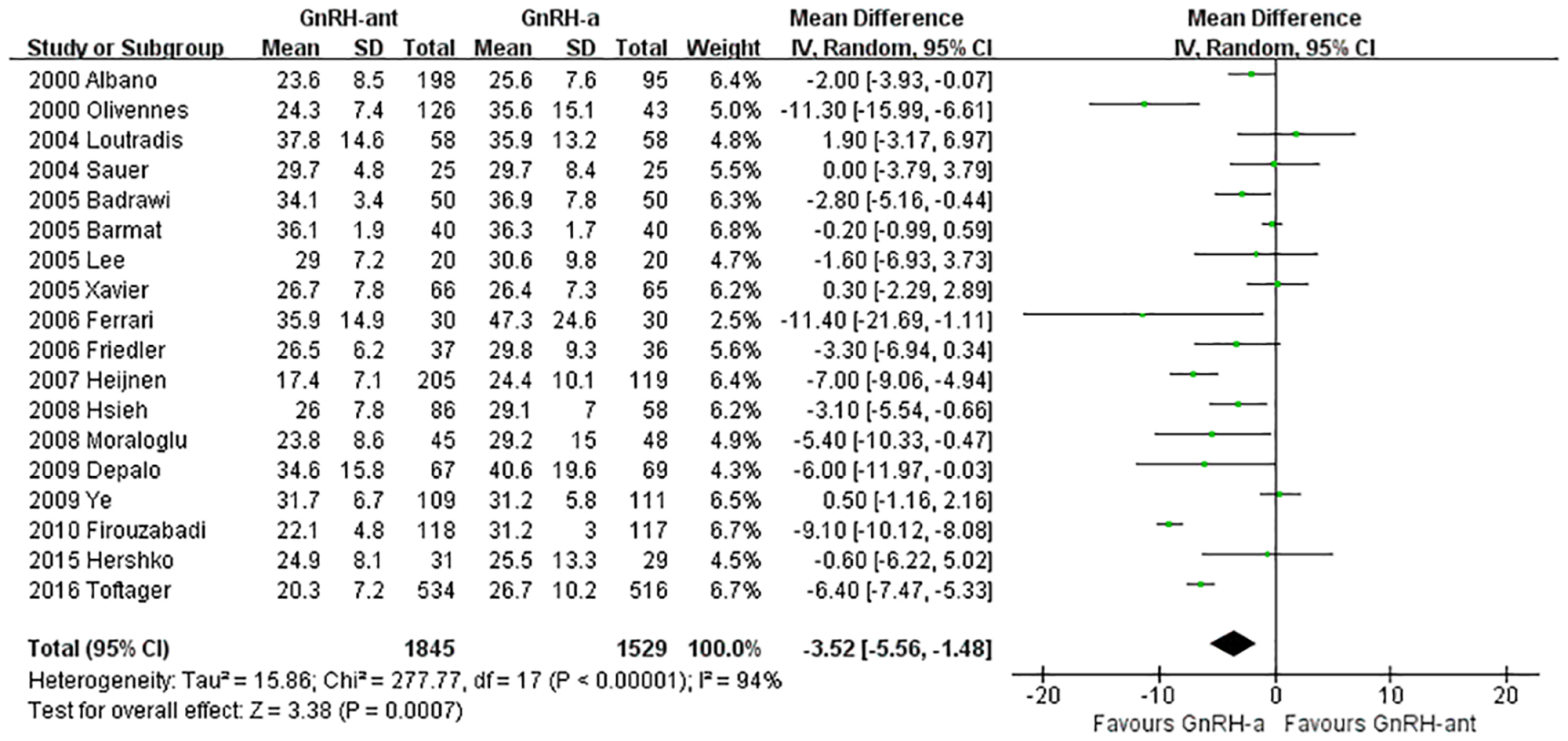
Forest plot comparing the Gn dosage per woman randomized between the GnRH-ant group and the GnRH-a long-protocol group.

A total of 18 studies [8, 14, 16, 21–27, 31–36, 40, 41](3,424 cases) were included in the Gn dosage meta-analysis. There was heterogeneity (P<0.00001, I^2^ = 94%) among the trials; therefore, the random-effects model was used for the meta-analysis. The results indicated that the Gn dosage was statistically significantly less in the GnRH-ant group than in the GnRH-a long-protocol group (MD [95% CI] = -3.52 [-5.56, -1.48], P = 0.0007, Fig 6).

#### The endometrial thickness on the day of HCG administration

**Fig 7:**
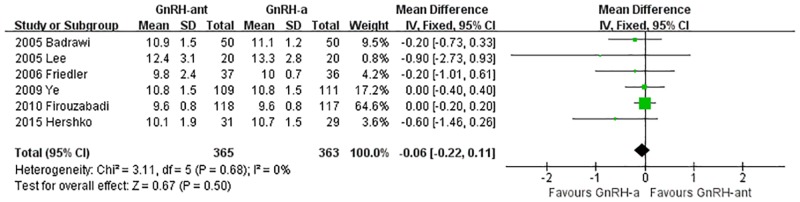
Forest plot comparing the endometrial thickness on the day of HCG administration per woman randomized between the GnRH-ant group and the GnRH-a long-protocol group.

A total of 6 studies [23, 24, 27, 35, 36, 40](698 cases) were included in the meta-analysis of endometrial thickness on the day of HCG administration. There was no heterogeneity (P = 0.68, I^2^ = 0%) among the trials; therefore, the fixed-effects model was used for the meta-analysis. The results indicated that there was no statistically significant difference in the endometrial thickness on the day of HCG administration between the GnRH-ant group and the GnRH-a long-protocol group (MD [95% CI] = -0.06 [-0.22, 0.11], P = 0.5, Fig 7).

#### The E2 level on the day of HCG administration

**Fig 8:**
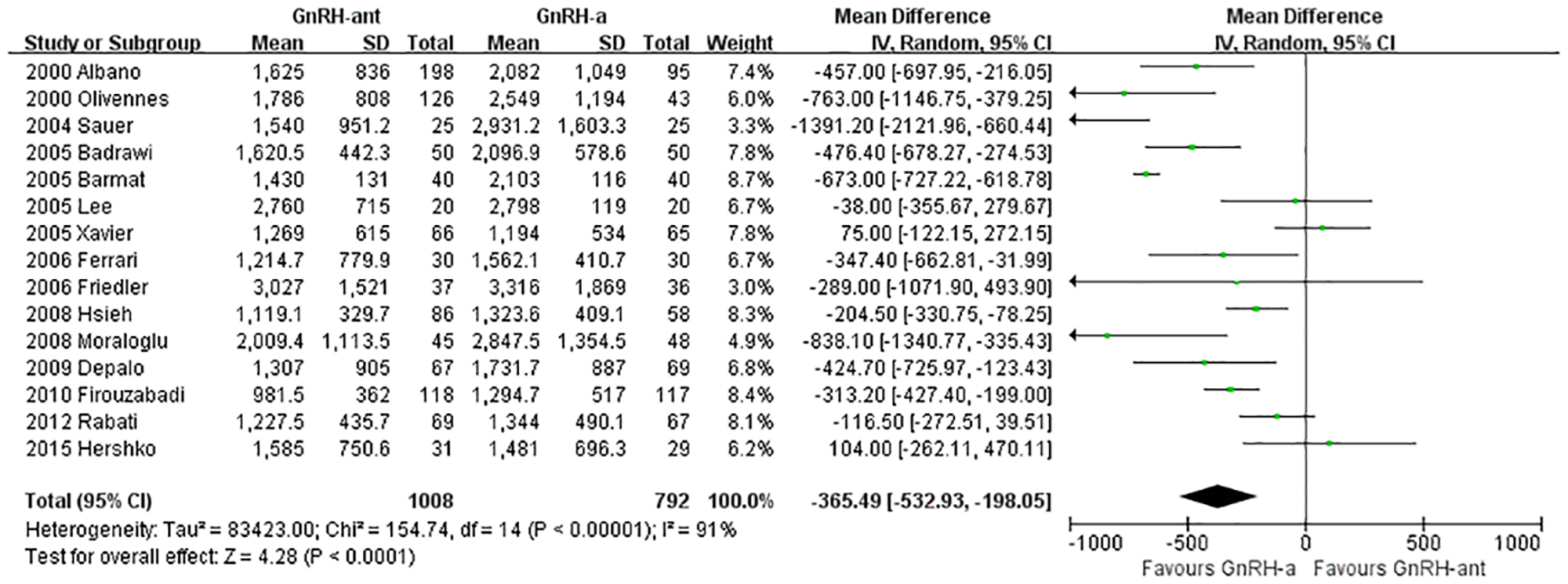
Forest plot comparing the E2 level on the day of HCG administration per woman randomized between the GnRH-ant group and the GnRH-a long-protocol group.

A total of 15 studies [8, 14, 16, 22–27, 32–34, 36, 39, 40](1,770 cases) were included in the E2 level on the day of HCG administration meta-analysis. There was heterogeneity (P<0.00001, I^2^ = 91%) among the trials; therefore, the random-effects model was used for the meta-analysis. The results indicated that the E2 level on the day of HCG administration was statistically significantly lower in the GnRH-ant group than in the GnRH-a long-protocol group (MD [95% CI] = -365.49 [-532.93, -198.05], P<0.0001, Fig 8).

#### The number of oocytes retrieved

**Fig 9:**
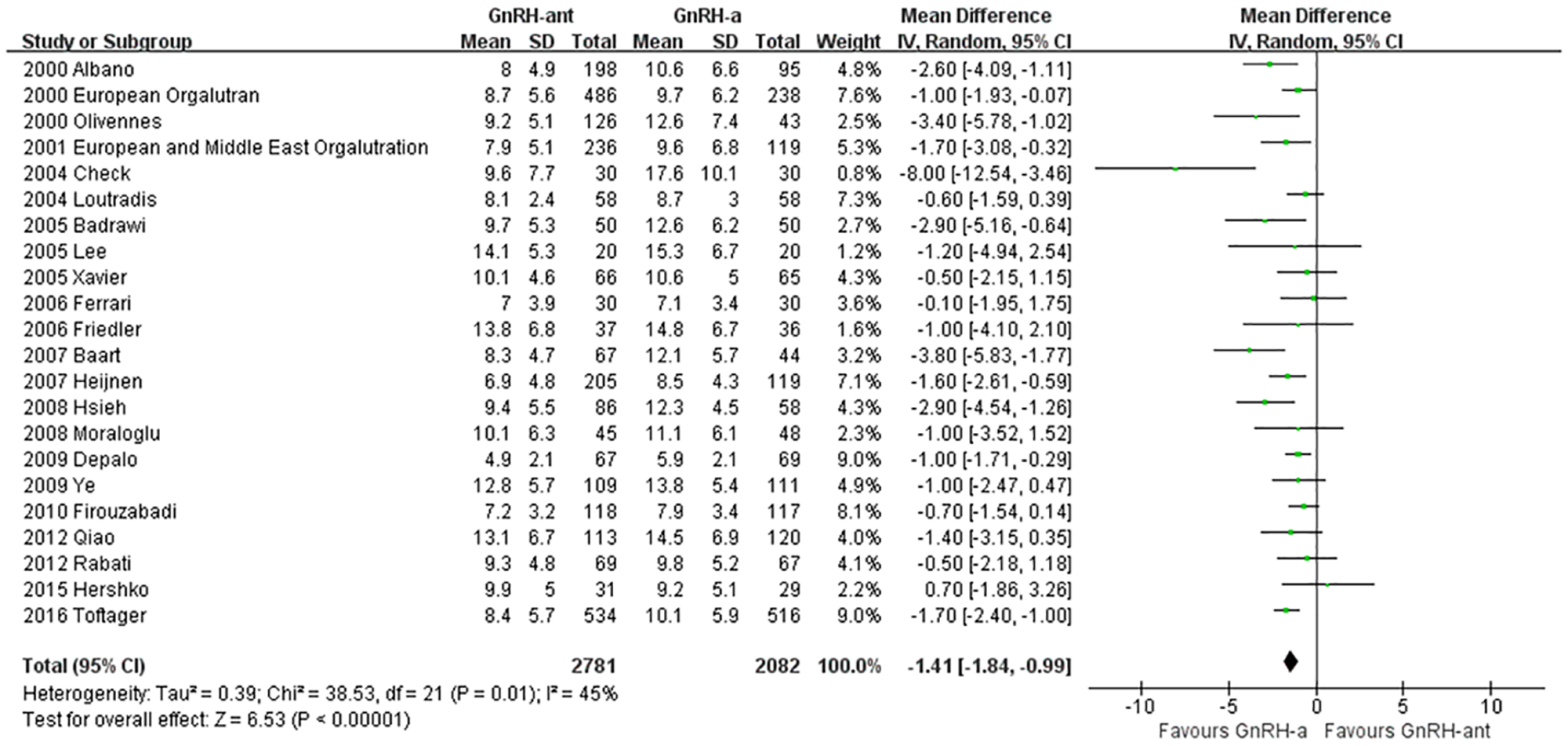
Forest plot comparing the number of oocytes retrieved per woman randomized between the GnRH-ant group and the GnRH-a long-protocol group.

A total of 22 studies [14–17, 20, 21, 23–27, 30–36, 38–41](4,919 cases) were included in the meta-analysis of the number of oocytes retrieved. There was heterogeneity (P = 0.01, I^2^ = 45%) among the trials; therefore, the random-effects model was used for the meta-analysis. The results indicated that the retrieved oocytes were statistically significantly fewer in the GnRH-ant group than in the GnRH-a long-protocol group (MD [95% CI] = -1.41 [-1.84, -0.99], P<0.00001, Fig 9).

#### The number of embryos obtained

**Fig 10:**
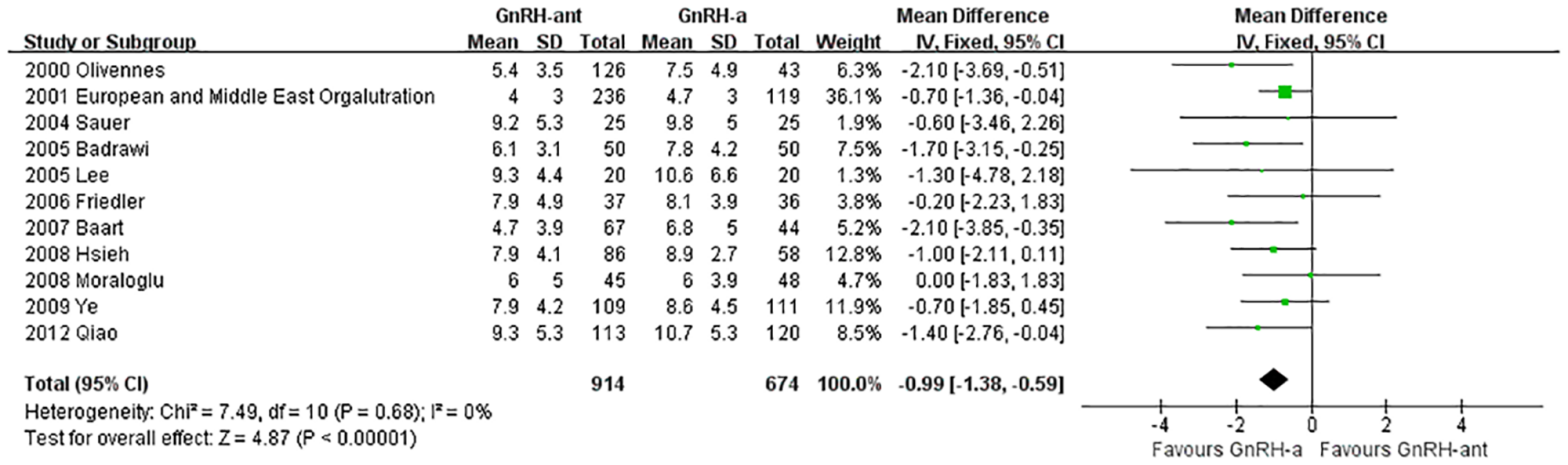
Forest plot comparing the number of embryos obtained per woman randomized between the GnRH-ant group and the GnRH-a long-protocol group.

A total of 11 studies [16, 17, 22–24, 27, 30, 32, 33, 35, 38](1,588 cases) were included in the meta-analysis of the number of embryos obtained. There was no heterogeneity (P = 0.68, I^2^ = 0%) among the trials; therefore, the fixed-effects model was used for the meta-analysis. The results indicated that the obtained embryos were statistically significantly fewer in the GnRH-ant group than in the GnRH-a long-protocol group (MD [95% CI] = -0.99 [-1.38, -0.59], P<0.00001, Fig 10).

### Outcome measures of safety

#### The incidence of OHSS

**Fig 11:**
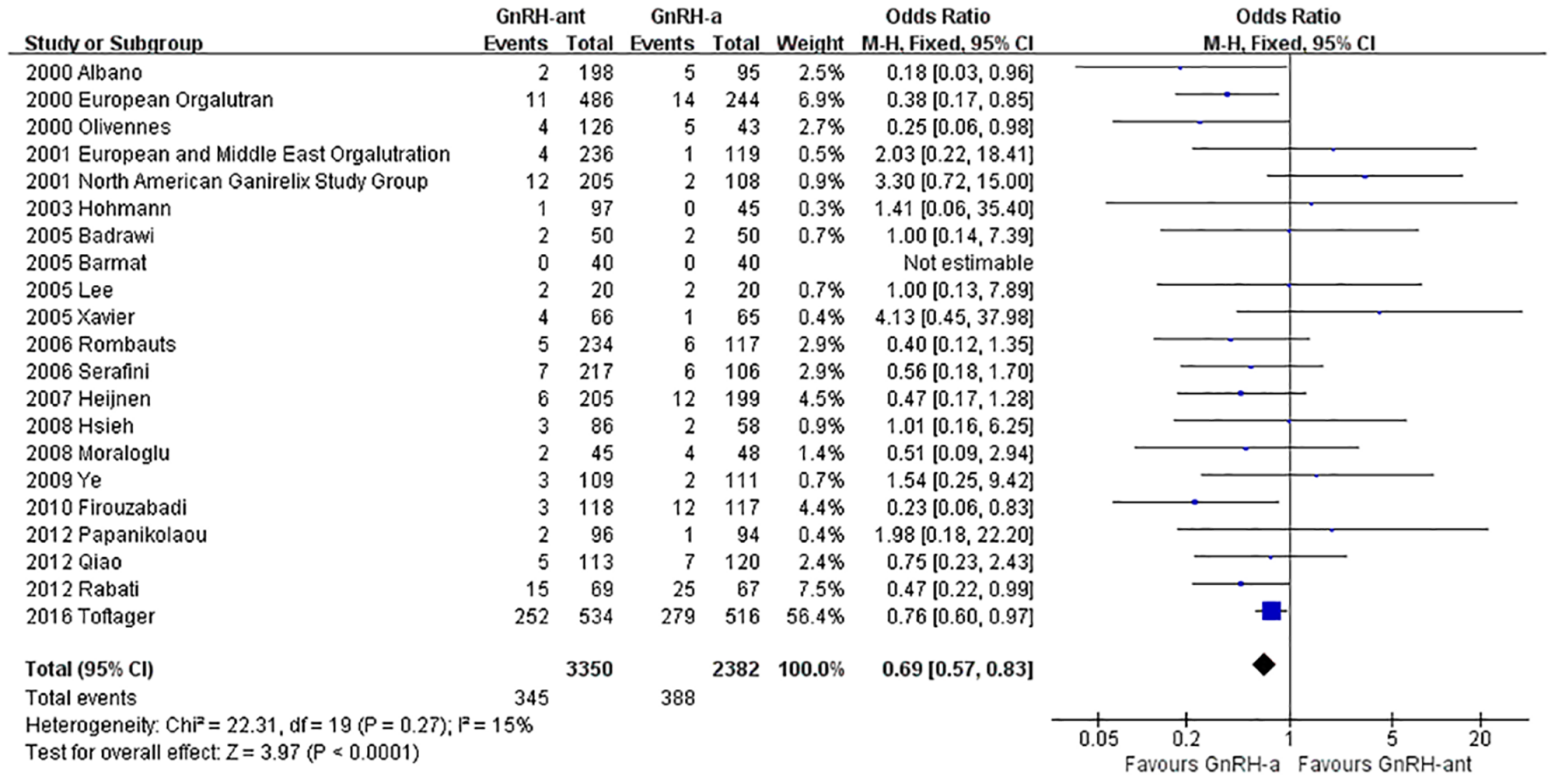
Forest plot comparing the incidence of OHSS per woman randomized between the GnRH-ant group and the GnRH-a long-protocol group.

A total of 21 studies [8, 14–19, 23–25, 28, 29, 31–33, 35–39, 41](5,763 cases) were included in the incidence of OHSS meta-analysis. There was no heterogeneity (P = 0.27, I^2^ = 15%) among the trials; therefore, the fixed-effects model was used for the meta-analysis. The results indicated that the incidence of OHSS was statistically significantly lower in the GnRH-ant group than in the GnRH-a long-protocol group (OR [95% CI] = 0.69 [0.57, 0.83], P<0.0001, Fig 11).

#### The miscarriage rate

**Fig 12:**
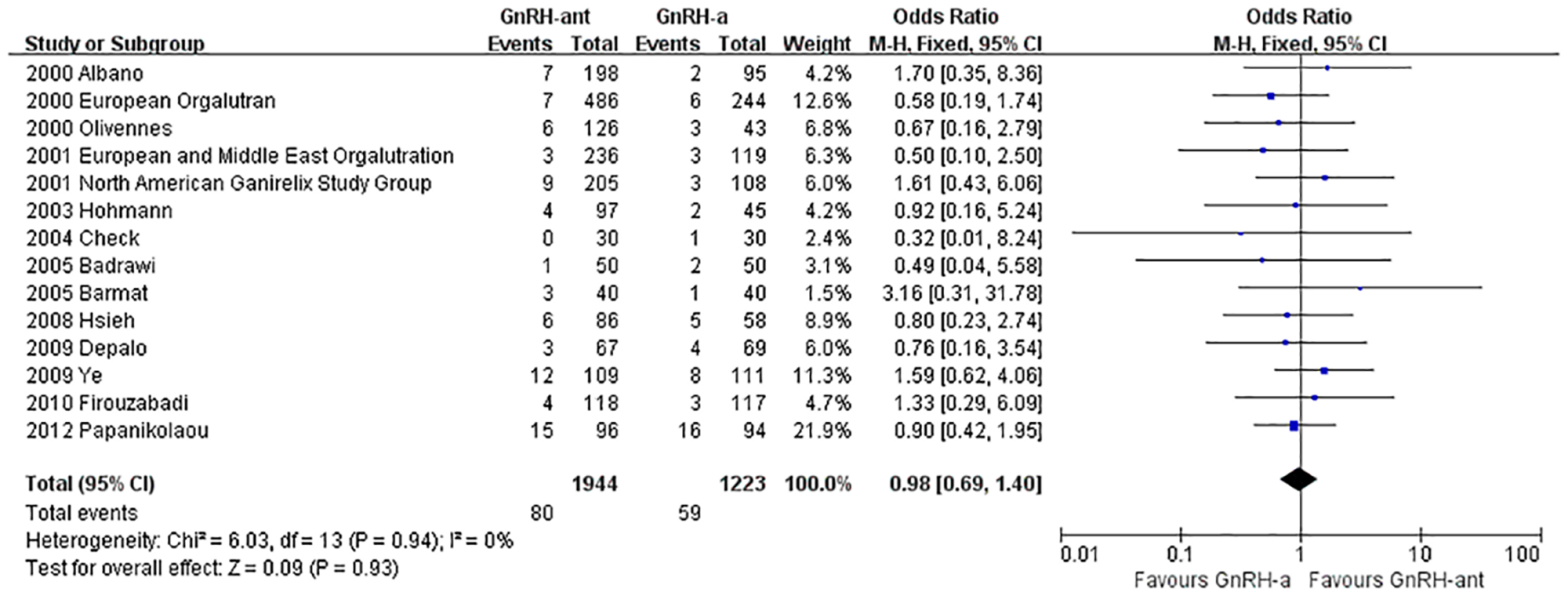
Forest plot comparing the miscarriage rate per woman randomized between the GnRH-ant group and the GnRH-a long-protocol group.

A total of 14 studies [8, 14–20, 23, 32, 34–37](3,198 cases) were included in the miscarriage rate meta-analysis. There was no heterogeneity (P = 0.94, I^2^ = 0%) among the trials; therefore, the fixed-effects model was used for the meta-analysis. The results indicated that there was no statistically significant difference in the miscarriage rate between the GnRH-ant group and the GnRH-a long-protocol group (OR [95% CI] = 0.98 [0.69, 1.40], P = 0.93, Fig 12).

#### The cycle cancellation rate

**Fig 13:**
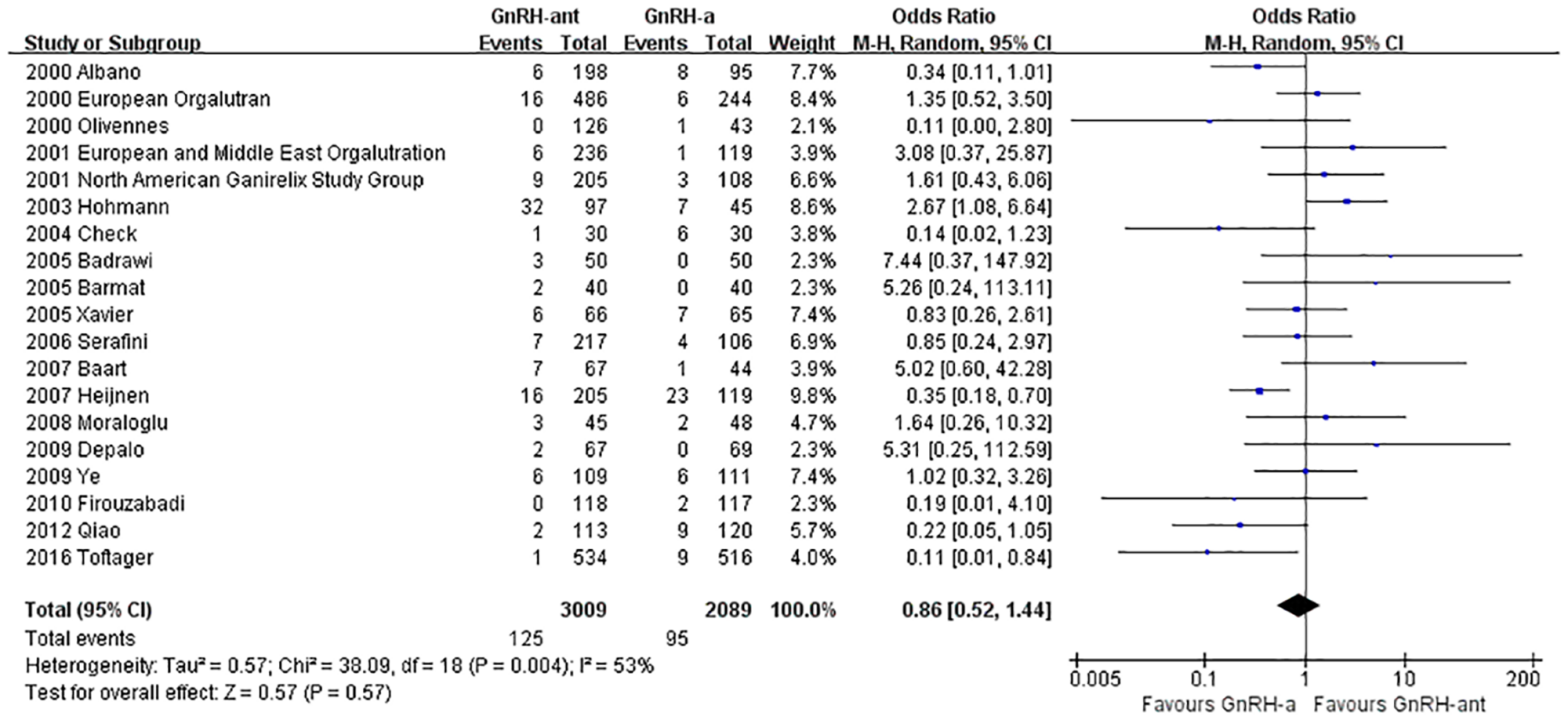
Forest plot comparing the cycle cancellation rate per woman randomized between the GnRH-ant group and the GnRH-a long-protocol group.

A total of 19 studies [8, 14–20, 23, 25, 29–31, 33–36, 38, 41](5,209 cases) were included in the cycle cancellation rate meta-analysis. There was heterogeneity (P = 0.004, I^2^ = 53%) among the trials; therefore, the random-effects model was used for the meta-analysis. The results indicated that there was no statistically significant difference in the cycle cancellation rate between the GnRH-ant group and the GnRH-a long-protocol group (OR [95% CI] = 0.86 [0.52, 1.44], P = 0.57, Fig 13).

### Sensitivity analysis and publication bias

The sensitivity analysis was performed by excluding the maximum weight studies [8, 17, 31, 36, 37, 41] in each outcome. The results showed that there was no influence on the pooled OR value; therefore, the outcomes are stable.

Begg’s funnel plots were symmetrical, and Begg’s tests had no significant publication bias (Begg’s test P>0.05, S1–S12 Figs).

## Discussion

### Summary of main results

Ovarian reserve can be tested by biochemical measures, such as basal follicle stimulating hormone (bFSH), anti-Mullerian hormone (AMH), and inhibin B level, as well as imaging measures, such as antral follicle count (AFC). The role of testing ovarian reserve is to help clinicians determine the appropriate protocol for individual patients, because ovarian reserve is the foundation of ovarian response to COH which may be vital for assisted reproductive technology (ART) outcome. Ovarian response can be divided into three types, low response, normal response, and high response. In 2011, the European Society of Human Reproduction and Embryology (ESHRE) standardized the definition of poor ovarian response [42], which usually occurs due to diminished ovarian reserve (DOR), and high response, which mainly refers to PCOS. However, there is no standard definition of normal ovarian reserve or normal response. Therefore, patients with normal ovarian reserve are included in our study to a certain extent based on a consensus.

Results of our meta-analysis mainly focused on the effectiveness and safety of GnRH antagonist protocol and GnRH agonist long protocol in COH. Regarding effectiveness, stimulation days, Gn dosage, E2 level on the day of HCG administration, and the number of oocytes retrieved, the embryos obtained were statistically significantly lower in GnRH-ant protocol than GnRH-a long protocol. The shorter stimulation period and lower Gn dosage in the GnRH-ant group may lead to the lower E2 level on the day of HCG administration, fewer retrieved oocytes, and fewer obtained embryos. However, regarding the main outcome measures of effectiveness, clinical pregnancy rate, ongoing pregnancy rate, and live birth rate had no significant differences between the two groups. The endometrial thickness on the day of HCG administration also showed no significant difference. This indicated that GnRH-ant protocol did not lead to a thicker endometrium on the day of HCG administration, and there was no difference in the reproductive outcome between the two groups. This means that even if the GnRH-ant protocol leads to fewer retrieved oocytes and fewer obtained embryos, this protocol does not reduce the chance of achieving a live birth. Moreover, GnRH-ant reduced the stimulation period and the Gn dosage, which is more convenient and cost-effective for patients.

The incidence of OHSS was statistically significantly lower in GnRH-ant protocol than in GnRH-a long protocol. This may because GnRH-ant protocol resulted in fewer retrieved oocytes, and a lower E2 level on the day of HCG administration, which had less possibility to induce OHSS than GnRH-a long protocol. No significant difference was found between the miscarriage rate, and the cycle cancellation rate between the two groups. Therefore, GnRH-ant protocol is safer because of the lower occurrence of OHSS without leading to miscarriage or cycle cancellation.

According to our results, GnRH-ant protocol can reduce the incidence of OHSS, faster and more cost-effectively, without affecting the reproductive outcome in normal ovarian reserve patients. Therefore, GnRH-ant protocol should be used as widely as GnRH-a protocol in these patients.

### Comparison with other studies

The recent Cochrane systematic review of 73 RCTs in 2016 has concluded that GnRH-ant protocol and GnRH-a protocol have equivalent live birth rates, while GnRH-ant protocol has a lower incidence of OHSS [11]. Their results are similar to ours. However, the patients with all types of ovarian reserve were included in their study, which covered DOR and PCOS.

A meta-analysis focusing on the supposed normal ovarian responders, including 23 RCTs (n = 3961), was conducted in 2014 [43]. The results indicated that the incidence of OHSS was significantly lower, whereas the ongoing pregnancy and live birth rates were similar in the GnRH antagonist and the standard long GnRH agonist protocols, which is similar to the results of our meta-analysis.

Another systematic review and meta-analysis of 22 RCTs (n = 3176) comparing GnRH-ant and GnRH-a reported the live birth as their main outcome measure [7]. No significant difference was present in the probability of live birth between the two GnRH analogs [OR = 0.86; 95% CI = 0.72 to 1.02]. This result remains stable in subgroup analysis that ordered the studies by type of population studied, Gn type used for stimulation, type of agonist protocol used, type of agonist used, type of antagonist protocol used, type of antagonist used, presence of allocation concealment, presence of co-intervention, and the way the information on live birth was retrieved. However, their study also included all types of ovarian reserve.

Additionally, a meta-analysis of four RCTs (n = 874) in 2008 mainly measured ongoing pregnancy rate [44]. The ongoing pregnancy rate per randomized woman was not found to be significantly different between patients with and without oral contraceptive pill (OCP) pretreatment (OR: 0.74, 95% CI: 0.53 to 1.03). Duration of Gn stimulation (WMD: 1.41 days, 95% CI: 1.13 to 1.68) and Gn consumption (WMD: 542 IU, 95% CI: 127 to 956) were significantly increased after OCP pretreatment. No significant differences were observed regarding the number of retrieved oocytes. However, the patients included in their study were also not classified by ovarian reserve.

### Limitations of this study

First of all, this meta-analysis lacked a uniform definition of normal ovarian reserve, which made it difficult to define inclusion and exclusion criteria, and the studies included may be not strict enough. Second, although the random-effects model was used to minimize the heterogeneity, it cannot be abolished. The heterogeneity may relate to the size of studies that ranged from 40 to 1,050 patients, and to the time span between 2000 and 2016. Additionally, the quality of reported studies was uneven, which may also lead to heterogeneity. The methods of randomization, concealment, and blinding were unclear in some included studies, some of which also had incomplete and selective outcome data. Third, although comprehensive searches were undertaken to ensure that all eligible studies were included, there is still the possibility that some potentially eligible studies were left out. All of these factors may lead to bias.

### Implications

The GnRH-ant protocol is a short, convenient, and cost-effective protocol in COH among patients with normal ovarian reserve, which can also reduce the incidence of OHSS without affecting the live birth rate when compared to GnRH-a long protocol. When referring to patients with normal ovarian reserve, in future clinical practice, GnRH-ant protocol may also be a good choice for COH. Moreover, a uniform definition for ovarian reserve, especially for normal ovarian reserve, should be standardized as soon as possible. RCTs of GnRH-ant protocol and GnRH-a long protocol in patients with different ovarian reserve are still lacking. And the study design of future RCTs should be more rigorous according to the CONSORT statement [45].

## Conclusion

In conclusion, our meta-analysis demonstrated that GnRH antagonist protocol substantially decreased the incidence of OHSS without influencing the pregnancy rate and live birth rate compared to GnRH agonist long protocol among patients with normal ovarian reserve. Therefore, for patients with normal ovarian reserve, GnRH-ant protocol should play a more important role in COH when clinicians are making individualizing and optimizing treatment decisions.

## Supporting information

S1 Table Quality assessment of studies. (DOC)

S1 PRISMA Checklist PRISMA 2009 checklist. (DOC)

S1 Fig Begg’s funnel plot and Egger’s test comparing the clinical pregnancy rate between the GnRH-ant group and the GnRH-a long-protocol group. (TIF)

S2 Fig Begg’s funnel plot and Egger’s test comparing the ongoing pregnancy rate between the GnRH-ant group and the GnRH-a long-protocol group. (TIF)

S3 Fig Begg’s funnel plot and Egger’s test comparing the live birth rate between the GnRH-ant group and the GnRH-a long-protocol group. (TIF)

S4 Fig Begg’s funnel plot and Egger’s test comparing the number of stimulation days between the GnRH-ant group and the GnRH-a long-protocol group. (TIF)

S5 Fig Begg’s funnel plot and Egger’s test comparing the Gn dosage between the GnRH-ant group and the GnRH-a long-protocol group. (TIF)

S6 Fig Begg’s funnel plot and Begg’s test comparing the endometrial thickness on the day of HCG administration between the GnRH-ant group and the GnRH-a long-protocol group. (TIF)

S7 Fig Begg’s funnel plot and Egger’s test comparing the E2 level on the day of HCG administration between the GnRH-ant group and the GnRH-a long-protocol group. (TIF)

S8 Fig Begg’s funnel plot and Egger’s test comparing the number of oocytes retrieved between the GnRH-ant group and the GnRH-a long-protocol group. (TIF)

S9 Fig Begg’s funnel plot and Egger’s test comparing the number of embryos obtained between the GnRH-ant group and the GnRH-a long-protocol group. (TIF)

S10 Fig Begg’s funnel plot and Egger’s test comparing the incidence of OHSS between the GnRH-ant group and the GnRH-a long-protocol group. (TIF)

S11 Fig Begg’s funnel plot and Egger’s test comparing the miscarriage rate between the GnRH-ant group and the GnRH-a long-protocol group. (TIF)

S12 Fig Begg’s funnel plot and Egger’s test comparing the cycle cancellation rate between the GnRH-ant group and the GnRH-a long-protocol group. (TIF)
