# Supplementary material for: Comparisons of GnRH antagonist protocol versus GnRH agonist long protocol in patients with normal ovarian reserve: A systematic review and meta-analysis
Source: PLoS One. 2017 Apr 24;12(4):e0175985. doi: 10.1371/journal.pone.0175985 (PMC5402978; doi:10.1371/journal.pone.0175985)
Supplement: S1 Table — (DOC) [file pone.0175985.s001.doc]

| Figure S2. Quality Assessment of Studies | | | | | | |
| --- | --- | --- | --- | --- | --- | --- |
| Source | Adequate Sequence Generation | Allocation Concealment | Blinding | Incomplete Outcome Data Addressed | Selective Outcome Reporting Avoided | Other Sources of Bias |
| Albano et al. 2000 | Unclear | Yes | No | Yes | Yes | Unclear |
| European Orgalutran. 2000 | Yes | Yes | No | Yes | Unclear | No |
| Olivennes et al. 2000 | Unclear | Unclear | No | Unclear | Yes | No |
| European and Middle East Orgalutration. 2001 | Yea | Yes | No | Yes | Yes | Unclear |
| Fluker et al. 2001 | Yes | Yes | No | Yes | Yes | No |
| Hohmann et al.2003 | Yes | Yes | No | Yes | Yes | No |
| Check et al. 2004 | Unclear | Unclear | No | No | Yes | Unclear |
| Loutradis et al. 2004 | Yes | Unclear | No | Unclear | Yes | No |
| Sauer et al. 2004 | Yes | Unclear | No | Yes | Yes | No |
| Badrawi et al. 2005 | Unclear | Yes | No | Yes | Yes | No |
| Barmat et al. 2005 | Yes | Yes | No | Yes | Yes | No |
| Lee et al. 2005 | Unclear | Unclear | Unclear | Unclear | Yes | No |
| Xavier et al. 2005 | Yes | Unclear | No | Unclear | Yes | No |
| Ferrari et al. 2006 | Yes | Unclear | Unclear | Yes | Unclear | No |
| Friedler et al. 2006 | Unclear | Unclear | No | Yes | Yes | No |
| Rombauts et al. 2006 | Unclear | Yes | No | Unclear | Yes | No |
| Serafini et al. 2006 | Yes | Unclear | Unclear | Unclear | No | No |
| Baart et al. 2007 | Yes | Yes | Yes | Yes | Yes | No |
| Heijnen et al. 2007 | Yes | Yes | No | Yes | Yes | No |
| Hsieh et al. 2008 | Uncear | Unclear | Unclear | Yes | Yes | Unclear |
| Moraloglu et al. 2008 | Yes | Yes | Unclear | Unclear | Yes | No |
| Depalo et al. 2009 | Yes | Unclear | Yes | Yes | Yes | No |
| Ye et al. 2009 | Yes | Unclear | No | Yes | Yes | No |
| Firouzabadi et al. 2010 | Yes | Yes | No | Yes | Yes | No |
| Papanikolaou et al. 2012 | Yes | Yes | Unclear | Yes | Yes | No |
| Qiao et al. 2012 | Unclear | Unclear | Unclear | Yes | Unclear | No |
| Rabati et al. 2012 | Unclear | Unclear | Unclear | Yes | Yes | No |
| Hershko et al. 2015 | Unclear | Yes | Unclear | Unclear | Unclear | No |
| Toftager et al. 2016 | Yes | Yes | No | Yes | Yes | No |
