# Supplementary figures and images for: Comparisons of GnRH antagonist protocol versus GnRH agonist long protocol in patients with normal ovarian reserve: A systematic review and meta-analysis
Source: PLoS One. 2017 Apr 24;12(4):e0175985. doi: 10.1371/journal.pone.0175985 (PMC5402978; doi:10.1371/journal.pone.0175985)

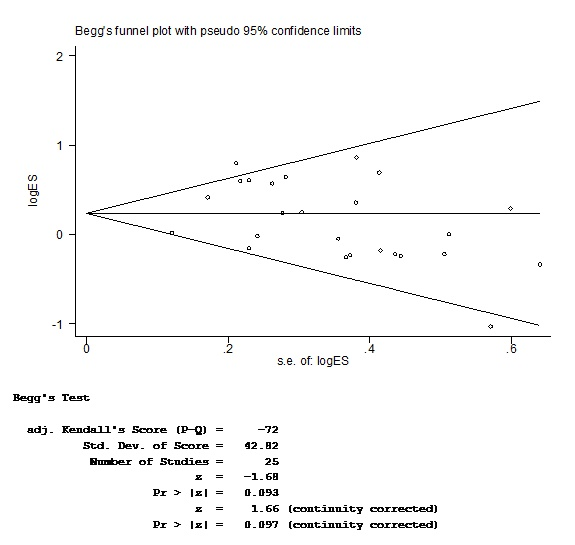

Supplement: S1 Fig — (TIF) [file pone.0175985.s003.tif]

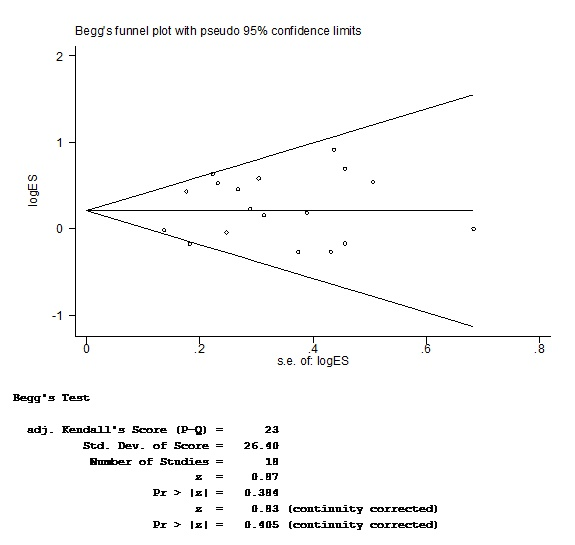

Supplement: S2 Fig — (TIF) [file pone.0175985.s004.tif]

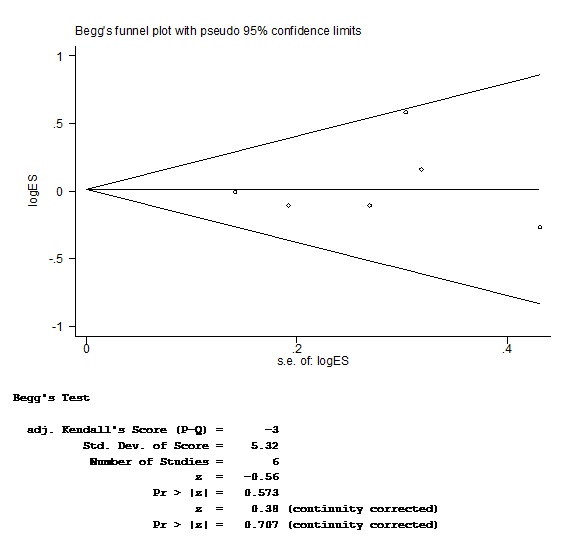

Supplement: S3 Fig — (TIF) [file pone.0175985.s005.tif]

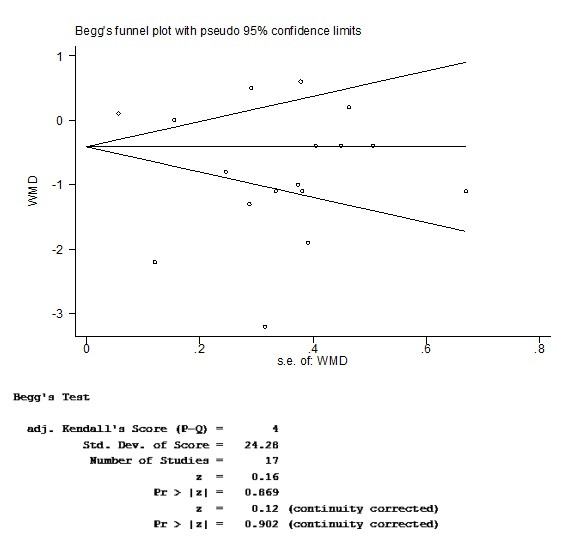

Supplement: S4 Fig — (TIF) [file pone.0175985.s006.tif]

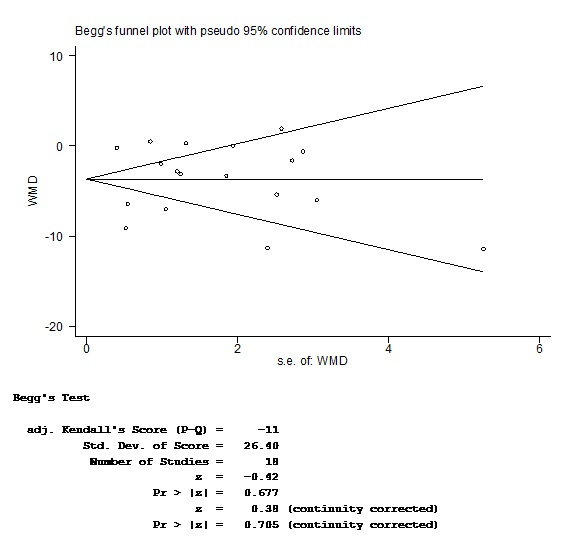

Supplement: S5 Fig — (TIF) [file pone.0175985.s007.tif]

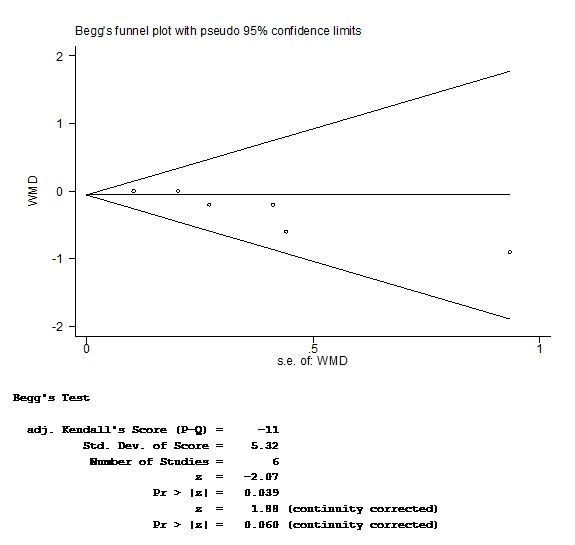

Supplement: S6 Fig — (TIF) [file pone.0175985.s008.tif]

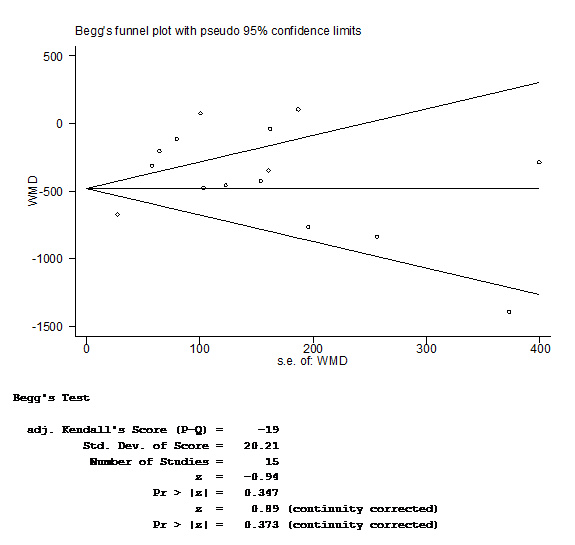

Supplement: S7 Fig — (TIF) [file pone.0175985.s009.tif]

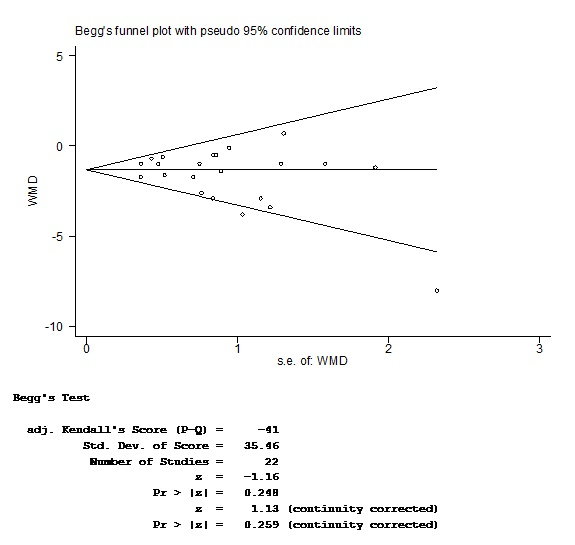

Supplement: S8 Fig — (TIF) [file pone.0175985.s010.tif]

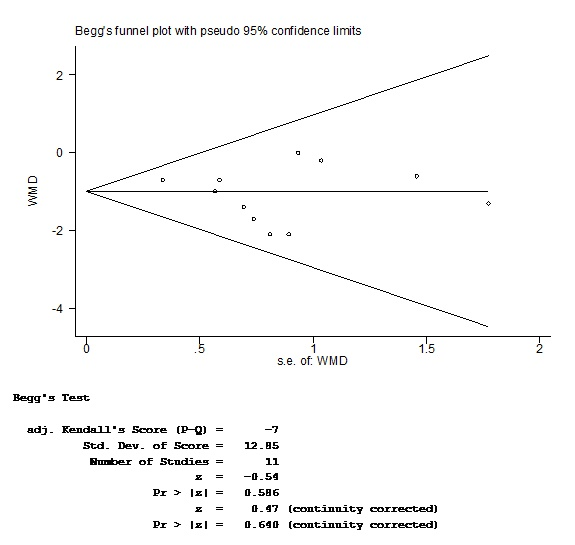

Supplement: S9 Fig — (TIF) [file pone.0175985.s011.tif]

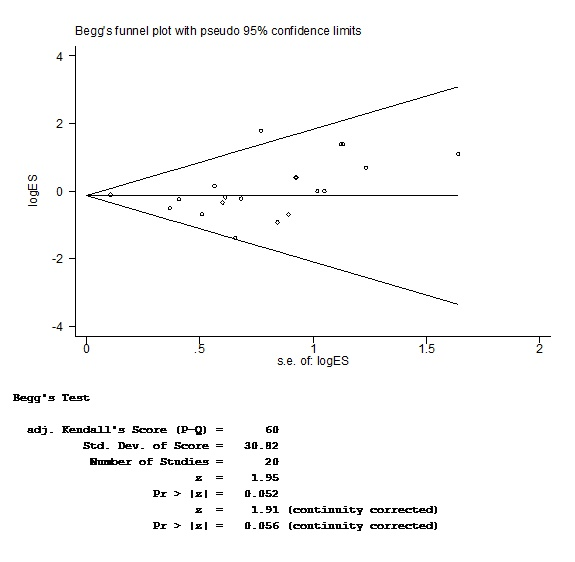

Supplement: S10 Fig — (TIF) [file pone.0175985.s012.tif]

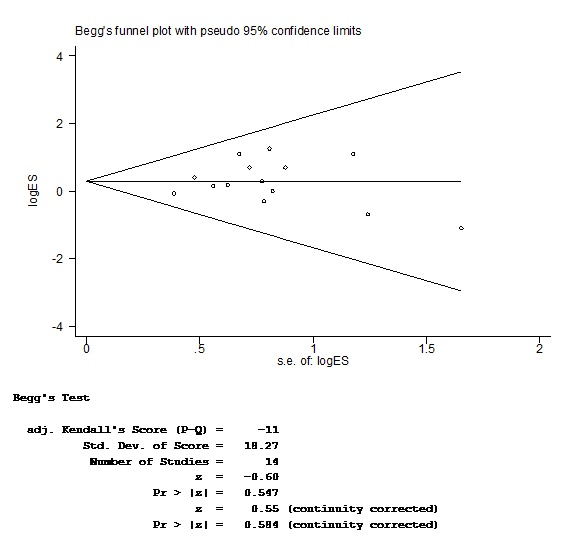

Supplement: S11 Fig — (TIF) [file pone.0175985.s013.tif]

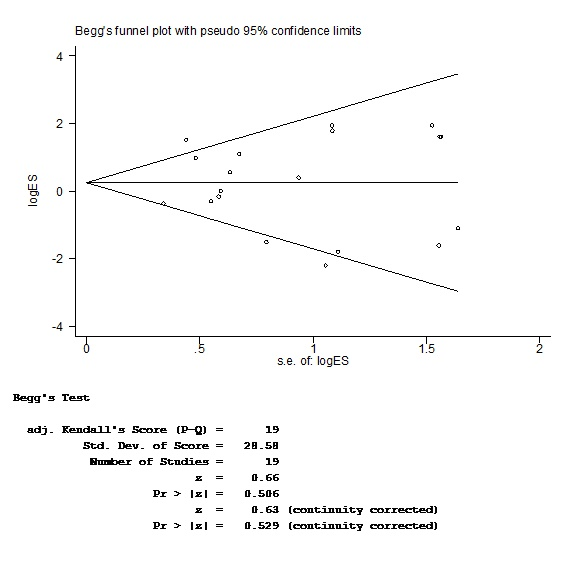

Supplement: S12 Fig — (TIF) [file pone.0175985.s014.tif]
